# Analysing ambiguities in trypanosomatids taxonomy by barcoding

**DOI:** 10.1590/0074-02760200504

**Published:** 2020-06-19

**Authors:** Carolina Boucinha, Amanda R Caetano, Helena LC Santos, Raphael Helaers, Miikka Vikkula, Marta Helena Branquinha, André Luis Souza dos Santos, Philippe Grellier, Karina Alessandra Morelli, Claudia Masini d‘Avila-Levy

**Affiliations:** 1 Fundação Oswaldo Cruz-Fiocruz, Instituto Oswaldo Cruz, Laboratório de Estudos Integrados em Protozoologia, Coleção de Protozoários da Fiocruz, Rio de Janeiro, RJ, Brasil; 2 University of Louvain, de Duve Institute, Laboratory of Human Molecular Genetics, Brussels, Belgium; 3 Universidade Federal do Rio de Janeiro, Instituto de Microbiologia Paulo de Góes, Rio de Janeiro, Brasil; 4 Muséum National d‘Histoire Naturelle, Unité Molécules de Communication et Adaptation des Microorganisme, Paris, France; 5 Universidade do Estado do Rio de Janeiro, Instituto de Biologia Roberto Alcântara Gomes, Departamento de Ecologia, Rio de Janeiro, RJ, Brasil

**Keywords:** barcode, barcoding, Kinetoplastea, taxonomy, Trypanosomatida, Trypanosomatidae, voucher

## Abstract

**BACKGROUND:**

Biodiversity screens and phylogenetic studies are dependent on reliable DNA sequences in public databases. Biological collections possess vouchered specimens with a traceable history. Therefore, DNA sequencing of samples available at institutional collections can greatly contribute to taxonomy, and studies on evolution and biodiversity.

**METHODS:**

We sequenced part of the glycosomal glyceraldehyde phosphate dehydrogenase (gGAPDH) and the SSU rRNA (V7/V8) genes from 102 trypanosomatid cultures, which are available on request at www.colprot.fiocruz.br.

**OBJECTIVE:**

The main objective of this work was to use phylogenetic inferences, using the obtained DNA sequences and those from representatives of all Trypanosomatidae genera, to generate phylogenetic trees that can simplify new isolates screenings.

**FINDINGS:**

A DNA sequence is provided for the first time for several isolates, the phylogenetic analysis allowed the classification or reclassification of several specimens, identification of candidates for new genera and species, as well as the taxonomic validation of several deposits.

**MAIN CONCLUSIONS:**

This survey aimed at presenting a list of validated species and their associated DNA sequences combined with a short historical overview of each isolate, which can support taxonomic and biodiversity research and promote culture collections.

The class Kinetoplastea is a noted group of protists that has one or two flagella, emerging from a flagellar pocket and that may exist in nature either as free-living species or as parasites of vertebrates, invertebrates or plants. The class is defined by the presence of a characteristic structure, the kinetoplast, a highly condensed DNA from a single mitochondrion that ramifies throughout the cell body.^1^

The family Trypanosomatidae encompasses members, which are the causative agents of severe human diseases that are mainly transmitted by an insect vector, such as Chagas disease (caused by *Trypanosoma cruzi*), sleeping sickness (caused by *Trypanosoma brucei sensu lato*) and the various forms of cutaneous and visceral leishmaniasis (caused by *Leishmania* spp.).^2^ In addition, there are currently 18 formally described genera that are generically known as insect trypanosomatids, because they are thought to have the life cycle restricted to an insect host, namely: *Angomonas*, *Blastocrithidia*, *Blechomonas*, *Borovskyia*, *Crithidia*, *Herpetomonas*, *Jaenimonas*, *Kentomonas*, *Lafontella*, *Leptomonas*, *Lotmaria*, *Novymonas*, *Paratrypanosoma*, *Rhynchoidomonas*, *Sergeia*, *Strigomonas*, *Wallacemonas* and *Zelonia*. (reviewed by^3^ and new genera described in^4^ ^,^ ^5^ ^,^ ^6^ ^,^ ^7^ ^,^ ^8^). Finally, there is one genus, *Phytomonas*, which alternates its life cycle between a plant and an insect.^9^ ^,^ ^10^

In a historical perspective, trypanosomatids taxonomy and diversity have been completely revolutionised by the so-called “molecular era”. For instance, from 1966 to 1990, only one new genus has been described,^1^ ^,^ ^11^ after the 90’s, 14 new genera were described, with a clear increase of genera description in the last years.^4^ ^,^ ^5^ ^,^ ^6^ ^,^ ^7^ ^,^ ^8^ ^,^ ^12^ This augment in the number of described taxa is not a simple consequence of more field expeditions and sample collection, it also reflects taxonomic revisions and proposals of new taxa to better reflect the phylogeny of previously described trypanosomatid isolates, whose true diversity has been hidden by traditional taxonomy approaches.^3^ ^,^ ^8^ ^,^ ^13^

It is clear nowadays that trypanosomatid taxonomy, previously strongly based on morphotypes, host specificity or site/mode of host colonisation, must also be supported by phylogenetic inferences. To this end, glycosomal glyceraldehyde-3-phosphate dehydrogenase (gGAPDH) and the V7/V8 variable region of the 18S small subunit ribosomal RNA (SSU) have been widely used and have been advocated as barcodes for trypanosomatid taxonomy, while the spliced leader (SL) RNA and the internal transcribed spacer (ITS) region of the ribosomal RNA are suitable to analyse strain or isolate diversity.^3^ ^,^ ^8^ ^,^ ^14^ The use of these markers allows the identification of species through comparison with sequences available in public databases. However, it is not uncommon that the public databases lack one or more of the aforementioned molecular markers, or even worse, the associated data of the sequenced organism is imprecise, incomplete, outdated or even incorrect. Therefore, culture collections with vouchered species and associated information are of outmost importance to pave the road for trypanosomatid taxonomy.^15^

The Fiocruz Protist Culture Collection (Fiocruz-COLPROT, www.colprot.fiocruz.br) has more than 400 live specimens of protists from several families, most of them representatives of the Kinetoplastea class, mainly members of the Trypanosomatidae family. This collection is constantly enriched with deposits from various collaborators, as well as field expeditions performed by its research team. To ensure reliable management of a culture collection is extremely important because it greatly increases the chances of sample availability and preservation for future generations.^15^

Here, we sequenced gGAPDH and SSU (region V7/V8) to provide an unequivocal molecular signature to 102 trypanosomatid specimens that compose part of the COLPROT catalogue. In addition, a detailed revision of the archived information regarding sample precedency, host, place and year of isolation and any relevant associated information was detailed, revised and made public. Our data led to the reclassification of several specimens based on recent taxonomic revisions and phylogenetic inferences, the identification of candidates to new genus and species, as well as the confirmation of the molecular identity of several deposits. Overall, this “molecular certification” offers to the scientific community a broader view of the family Trypanosomatidae.

## MATERIALS AND METHODS

*Chemicals* - Media constituents, reagents used in electrophoresis, buffer components, agarose and all other reagents are of analytical grade or superior. The following kits were purchased from Promega: Wizard® Genomic DNA Purification kit and Wizard® SV Gel and PCR Clean-Up System. GelRed™ fluorescent DNA stain was obtained from Biotium. The BigDye® Terminator v3.1 Cycle Sequencing was obtained from Life Technologies. Foetal bovine serum (FBS) was purchased from Cultilab and heat inactivated.

*Trypanosomatid cultures* - All organisms examined in this study are cryopreserved at Fiocruz Protist Culture Collection (COLPROT) (http://colprot.fiocruz.br). The host, geographical origin, isolator, depositor and year of isolation are summarised in Supplementary data (Table I). The initial taxonomic identification used was the one provided by the original depositor, which are cataloged in the COLPROT records. Ampoules with the specimens were defrosted, and all its content was grown in biphasic medium NNN/LIT (Novy-MacNeal-Nicolle/Liver Infusion Tryptose) supplemented with 10% FBS. When necessary, cells were subcultured in LIT to reach logarithmic growth phase.

*DNA extraction, polymerase chain reaction (PCR) amplification and DNA sequencing* - Total DNA from at least 5 × 10^6^ cells was extracted from cultured trypanosomatids at mid-log growing phase using the Wizard® Genomic DNA Purification kit according the manufacturer’s protocol. The V7/V8 SSU rRNA (V7V8 SSU rRNA F 5’-CAC CCG CGG TAA TTC CAG C-3’ and V7V8 SSU rRNA R 5’-CTG AGA CTG TAA CCT CAA-3’) and the gGAPDH (gGAPDH F 5′- GGB CGC ATG GTS TTC CAG-3′ and gGAPDH R 5′- CCC CAC TCG TTR TCR TAC C -3′) genes were PCR-amplified using previously described PCR conditions with small modifications, when required.^12^ Briefly, both genes were PCR-amplified to approximately 800 bp fragments with the following program: V7V8 SSU rRNA - 94ºC, 300 s followed by 35 cycles of 30 s at 94ºC, 120 s at 52 to 60ºC, 120 s at 72ºC and a final extension period of 72ºC for 600 s; gGAPDH - 94ºC, 180 s followed by 30 cycles of 60 s at 94ºC, 120 s at 52 to 60ºC, 120 s at 72ºC and a final extension period of 72ºC for 600 s. The PCRs were subjected to reagents and temperature adjustments to amplify the region of each of the molecular markers used for each species analysed. The amplified DNA segments were visualised by staining with GelRed after electrophoresis separation in 1.5% agarose gel in a horizontal vessel, prepared at TBE solution (1.1 M Tris, 0.9 M Borate and 25 mM ethylenediamine tetra-acetic acid, pH 8.3) for 50 min at 90 V. PCR reaction products were purified by Wizard SV Gel and PCR Clean-Up System kit, and sequenced in both directions using the BigDye Terminator v3.1 cycle sequencing kit and loaded in the ABI 3730 Sequencing Platform at Fundação Oswaldo Cruz. Sequences were analysed and edited using the SeqMan software (DNASTAR software package, DNASTAR Inc., Madison, WI, USA).

*Molecular identification and phylogenetic analysis* - The generated sequences were compared with available trypanosomatid sequences in Genbank by using BLASTn search. For phylogenetic analyses using maximum likelihood (ML) or Bayesian (BI) inferences, the DNA sequences obtained in this study [Supplementary data **(Table I)**] or retrieved from Genbank [Supplementary data (Table II)] were aligned using multiple sequence alignment with high accuracy and high throughput (MAFFT) online server.^16^ The alignment was automatically trimmed using METAPIGA v.3.1.^17^ Three alignments were created: (i) *V7/V8*SSU rRNA sequences, (ii) gGAPDH sequences and (iii) concatenated sequences. The final dataset, which compiles sequences generated in this study and sequences retrieved from GenBank, contained 134 specimens and 410 nucleotide positions (V7/V8), 159 specimens and 720 nucleotide positions (gGAPDH) and 161 taxa and 1130 nucleotides (concatenated analysis). The evolutionary model (GTR+I+G - General Time Reversible with Invariant and Gamma distribution) was selected using Akaike criterion in METAPIGA v2.0. ML phylogenetic inference was performed in METAPIGA v2.0 with the selected model and 1,000 bootstrap replicates. Other parameters were used as default states. We also inferred BI to concatenated sequences tree (18S SSU and gGAPDH) using MrBayes v3.2.^18^ For this, we used GTR+I+G substitution model with 100.000 generations with trees sampled every 10.000 generations using chains, and 25% of the early sample trees were discarded.

*Historical research of deposits of Protist Collection* - For each COLPROT deposit used in this work, a historical survey was done through revising the Collection database, research on abstracts of conferences and, whenever possible or necessary, interview with the depositors. Data from this research fueled the internal database SICol (http://sicol.fiocruz.br/), and are available in Supplementary data (Tables I, III **)**, the updated curated species are listed in COLPROT on-line catalogue (http://colprot.fiocruz.br).

## RESULTS AND DISCUSSION

The phylogenetic analyses based on the concatenated gGAPDH and SSU rRNA sequences or on the independent gene sequences (data not shown) using either ML or BI analysis resulted in similar topologies (Figure). A total of 102 trypanosomatids cultures were sequenced for gGAPDH and/or for V7/V8 [Supplementary data (Table I)]. Through the combination of BLASTn searches, pair-to-pair distance and phylogenetic analyses, a reference sequence for each taxon was selected [Supplementary data (Table II)] and included in Supplementary data (Table I) together with the identity percentage to the DNA sequences generated herein. The sequenced specimens comprehended after taxonomy revision and update a total of ten genera (*Angomonas*, *Crithidia*, *Herpetomonas*, *Kentomonas*, *Lafontella*, *Leptomonas*, *Phytomonas*, *Strigomonas*, *Wallacemonas* and *Zelonia*) distributed in 38 species [Supplementary data (Table I)]. Four distinct outcomes arose from the present data: (i) isolate certified, i.e., the DNA sequencing of the available isolate matched the sequences available in Genbank and the available literature [Supplementary data (Table I)], (ii) isolate classification, i.e., the isolate was not assigned to species yet, (iii) isolate re-classification, i.e., the isolate was previously assigned to a species before the “molecular era” and it was reassigned to another taxon to better reflect the phylogeny, and (iv) new typing unit, when the DNA sequencing and the phylogenetic analysis support for a new genus/species proposition [Supplementary data (Table I)]. Due to extensive notes of data, we comment below only the taxa that deserves highlight or the description of their taxonomic historical assembly. All information about the hosts of COLPROT deposits can be found in Supplementary data (Table III), the deposits are mainly from Brazil and are distributed in four insect orders, while Hemiptera and Diptera are the most abundant.

**Figure:**
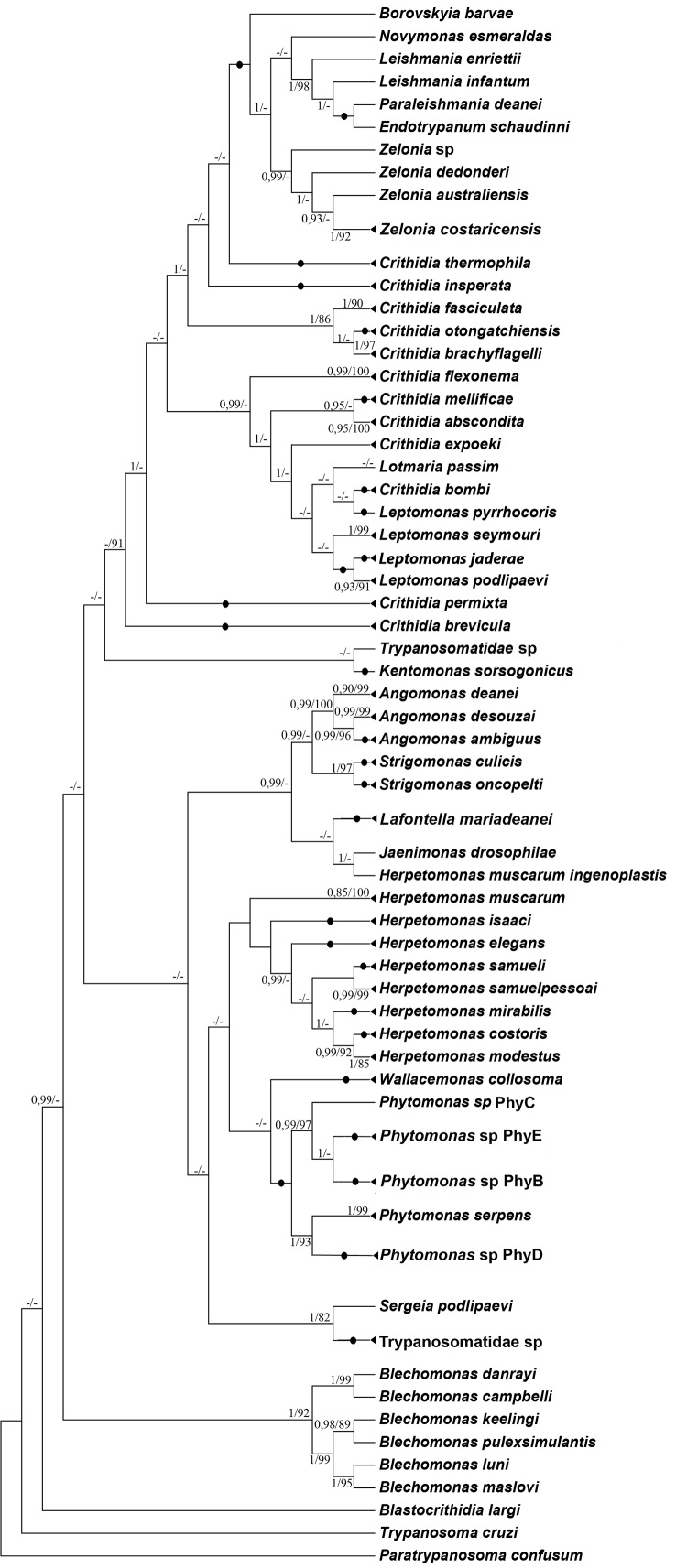
Molecular phylogenetic analysis by maximum likelihood (ML) and Bayesian (BI) inferences from concatenated data set. Phylogenetic tree of isolates studied in this work and Genbank sequences inferred by ML and BI analyses using *V7/V8SSU rRNA* region + *gGAPDH* concatenated set, with General Time Reversible model. A discrete Gamma distribution was used to model evolutionary rate differences among sites (six cat egories (+*G*, parameter = 0.5618)). The rate variation model allowed for some sites to be evolutionarily invariable (+*I*), 22.26% sites. The tree is rooted with *Paratrypanosoma confusum*. Numbers at nodes correspond respectively to BI and ML support values. The dash indicates support values below 80% from bootstraps derived from 1000 replicates, or distinct topology.

***Angomonas* spp.**

The genus *Angomonas* was validated by Teixeira and colleagues^12^ to allocate trypanosomatids harboring endosymbionts and presenting the choanomastigote form, but not related to the genus *Crithidia*.

*Angomonas deanei* - The material type for this species is the TCC 036E culture deposited in the Trypanosomatid Culture Collection of the University of São Paulo (TCC-USP), replacing the culture of the American Type Culture Collection (ATCC) 30255 (unavailable).^12^ TCC 036E and COLPROT044 are duplicates of *A. deanei*, which were described originally as *Crithidia deanei*.^19^ In 2015, Dr E Camargo donated TCC 036E to COLPROT (698), and DNA sequencing confirmed the expected molecular identity. The new deposit of old preserved samples from different sources are considered a proof of good practices in Culture Collections throughout the world.^15^

The isolate COLPROT212 was received in 1996 directly from the isolator, PM Faria-e-Silva, as *Herpetomonas roitmani*,^20^ which was originally described as *Crithidia roitmani*.^21^ This strain was later classified as *A. deanei* ^12^ COLPROT212 and TCC 080E represent duplicates of this isolate.

COLPROT226 was deposited in 1994 by JE Fiorini. The trypanosomatid was isolated in city of Alfenas/MG/BR and the source is questionable, since it is stated that it was isolated from a blackberry in the original records, but the cited reference refers to an isolate from *Cucurbita moschata*, a pumpkin typical for the Americas.^22^ Irrespective to which information corresponds to the actual source of the isolate, both represent fruits, and as such, this is the first report of isolation of *Angomonas* from a plant and adds more data to the discussion on host specificity in trypanosomatids. However, caution must be taken from any assumption since this represents an old specimen with doubts in its records.

*Angomonas desouzai* - Type material: Culture ATCC 50305, for which TCC 079E is the duplicate hapantotype deposited at TCC-USP.^12^ COLPROT109 is a duplicate of TCC 079E. The strain was isolated by Faria-e-Silva^21^ and later described as *Crithidia desouzai*. This strain was later moved to the *Angomonas* genus,^12^ which contains an endosymbiont and forms a strict clade with *Strigomonas*. An isolate of this species preserved at University of Ostrava was deposited at COLPROT in 2015 (COLPROT708) and revealed low genetic divergence for *gGAPDH* [Supplementary data (Table I)]. Also, during field expeditions done by Dr Jan Votýpka (Charles University, Prague), a strain was isolated from a fly (*Brachycera*) in 2015, in Angra dos Reis/RJ/Brazil (COLPROT704). These deposits had their taxonomic identification confirmed through the use of the molecular markers in this work.

The deposits of the species *Angomonas ambiguus* (COLPROT702) presented very low sequence divergence from Genbank reference strains [Supplementary data (Table I)] and had therefore the taxonomic status confirmed (Figure).

***Crithidia* spp.**

Classically, this genus would be easily distinguishable from another insect trypanosomatids through morphology. The choanomastigote morphotype is advocated to be easily recognisable in light microscopy, even for non-specialised observer. However, phylogenetic studies using *SSU rRNA* and *gGAPDH* clearly indicate that this genus is polyphyletic and together with the genus *Leptomonas* need a revision and reclassification, as clearly demonstrated in Figure.^12^ ^,^ ^14^ ^,^ ^23^ ^,^ ^24^ ^,^ ^25^

*Crithidia acantocephali* and *Crithidia flexonema* - The type strain for *C. flexonema* was deposited at ATCC (50211) by LS Diamond in 1960. The trypanosomatid was isolated in Ames, IA, USA, from the host *Gerris remiges*,^26^ which was later renamed to *Aquarius remiges* [Supplementary data (Table III)].^27^ COLPROT016 is a duplicate. Up to now, there was no reference sequence in Genbank for this species.

Another isolate without reference sequence in Genbank is *C. acantocephali*,^28^ which was isolated from *Acanthocephala femorata* [Supplementary data (Table III)], and deposited and preserved in ATCC, for which COLPROT042 is a duplicate. This, together with a previously unidentified deposit (COLPROT254) isolated in Brazil from *Zelus* sp., present high sequence identity to COLPROT016 [Supplementary data (Table I)] and formed a monophyletic group, as seen in Figure. Therefore, we suggest a synonymisation to the first described species, which is *C. flexonema*. Although the described year of isolation argues in favor of *C. acantocephali*, we took into consideration the year of publication (1960 for *C. flexonema* and 1961 for *C. acantocephali*). It is also interesting to note that the species display a high geographic dispersion and are capable of colonising very distinct hosts.

*Crithidia bombi, Crithidia expoeki, Lotmaria passim* and *Crithidia mellificae* - Trypanosomatids infecting honeybees have been poorly studied with molecular methods up to early 2000s. After the description of *C. mellificae*,^29^ it took forty years until molecular data for honeybee trypanosomatids became available. *C. mellificae* is a trypanosomatid parasite of *Apis mellifera* that was first described in Australian bees in 1967.^29^ Schmid-Hempel and Tognazzo^24^ restarted the discussion on trypanosomatid occurrence in honeybees and presented for the first time the molecular identity of the associated trypanosomatids.

The species *C. mellificae* represents the oldest described trypanosomatid from *A. mellifera*, and it has two available cultures deposited in ATCC (30254 and 30862). However, the lack of associated molecular data of these taxa together with the recent molecular screening of honeybee’s colonies resulted in assumptions that require considerations. In a wide honeybee microbiome screen, a trypanosomatid was repeatedly identified. The parasite was isolated, cultured and sequenced. Considering its choanomastigote shape and its host, it was assumed that it was a new isolate of *C. mellificae* and a molecular identity was assigned.

This isolate was afterwards named *C. mellificae* strain SF, and the whole genome was sequenced.^30^ Later, Cepero research group,^31^ while analysing honeybee colonies in Spain, compared the available *gGAPDH* sequences of *C. mellificae* isolates to the ones generated under their analysis, including *C. mellificae* ATCC 30254. It is clear that confusion was generated due to the deposit in Genbank of DNA sequences assumed to belong to *C. mellificae* without a molecular signature for the reference strain. This issue was clarified by Cepero^31^ and Schwarz^32^ research groups, who independently and almost simultaneously sequenced the available presumed *C. mellificae* strains, as well as the old available *C. mellificae* ATCC strains. Both authors identified considerable differences between the earlier described taxa and the more recent description of a trypanosomatid from honeybees. Schwarz and colleagues proposed to erect the genus *Lotmaria* ^32^ to accommodate the more recently described isolates from honeybees. COLPROT035 is a duplicate of ATCC 30254, and the *gGAPDH* and *V7/V8* sequences confirm its identity [Supplementary data (Table I)].

*C. bombi* was originally described morphologically^33^ and was later described based on classical molecular markers. This species has been described in bumble bees, has a worldwide distribution and is a very important pathogen of this insect.^24^ In fact, *C. bombi*, *C. mellificae* and *Crithidia expoeki* are important bees pathogens with impact on the insects’ fitness and survival. They represent a group of well-studied trypanosomatids due to the negative impact in honeybee populations, and in the economy. Clearly, the population decline causes serious consequences not only on honey production, but on the overall pollination process, with ecological and economic impacts. The neotype of *C. bombi* is represented by BJ08.085 strain, isolated in Movelier, district of Delémont, Switzerland. COLPROT683, COLPROT684 and COLPROT678 represent distinct isolates of this species.^24^ *C. expoeki* was isolated in the same host set, however, it presented a clear genetic divergence from *C. bombi.* The distinct clones of these three species can be differentiated based on ITS and cytochrome b sequences, while the species can be differentiated based on *gGAPDH* and *SSU rRNA* sequences.^24^ The hapantotype of *C. expoeki* is the isolate BJ08.175, recovered from *Bombus lucorum* in Röschenz, Switzerland, COLPROT679 is a duplicate, and COLPROT685-687 are isolates of the same species [Supplementary data (Table I)].

*Crithidia fasciculata* - The first technical name given to a mosquito flagellate was *C. fasciculata* Léger.^34^ Léger described the species as small flagellates, shaped like a barely corn truncate at the anterior end, which contains a funnel-like depression.^35^ Afterwards, the morphology proved to produce difficulties to assign new isolates to this species, and Wallace^35^ proposed the first taxonomic revision of monoxenic trypanosomatids. In this sense, the formerly described *Herpetomonas culicidrum* ^36^ was synonymised to *C. fasciculata*. This isolate is deposited at ATCC (11745), and COPROT048 is a duplicate. It was isolated from *Anopheles quadrimaculatus* [Supplementary data (Table III)]. This is the oldest preserved *C. fasciculata* strain with viable available cultures.

However, the strain Cf:C1, which has a poor traceable history, has been considered a reference sequence, and it has a publicly available genome. The strain was obtained from Dr Larry Simpson, University of California, Los Angeles, USA. The oldest reference refers to this strain as “The cells represent a clone of a *C. fasciculata* culture originally obtained from Dr Stuart Krassner. This clonal cell line has been growing in our laboratory for three years in Brain-Heart Infusion Medium”.^37^ Curiously, two extensive revisions of available trypanosomatid isolates failed to describe any *C. fasciculata* isolated by Kassner.^1^ ^,^ ^11^ Therefore, although rich in genomic information and being considered in the literature as a reference strain, the host, place of isolation and any other biological aspect regarding its occurrence in nature are unknown. For barcoding/taxonomy purposes, we compared the sequences generated for all *C. fasciculata* isolates with the available genome from Cf:C1 clone, as well as to the coding sequence of *gGAPDH* for the COLPROT048 (ATCC 11745) strain.^38^ COLPROT048, the oldest preserved culture, and COLPROT050, the second oldest culture, have low sequence divergence of *V7/V8* and *gGAPDH* markers to the Cf:C1 strain. In addition, as previously published, *Crithidia luciliae* (COLPROT053) should be synonymised to *C. fasciculata* [Figure and Supplementary data (Table I)].^39^ Notwithstanding, *Crithidia guilhermei* (COLPROT051)^39^ and *Crithidia ricardoi* (COLPROT184)^40^ also present very low sequence divergence and should all be considered isolates of a single species [Figure and Supplementary data (Table I)]*.* Furthermore, COLPROT213, isolated from a plant, and COLPROT606, isolated from a human patient, were also identified as isolates of *C. fasciculata* [Figure and Supplementary data (Table I)]. The molecular revision of these isolates shows for the first time that *C. fasciculata* is widespread, geographically and in host occurrence. It is reasonable to assume that further screening of either old samples or new isolates will reveal further the distribution of this species, particularly in the view that it can be found in plants, mammalian hosts^41^ and in a wide range of insects of the order Diptera [Supplementary data (Table III)].

*Crithidia thermophila* - The thermal resistance of monoxenous trypanosomatids has for a long time attracted the attention of researchers. As initially presumed, these trypanosomatids are unable to survive in a mammalian host, and as such, it was somewhat surprising that they can grow well in temperatures that are typical for blood-warm hosts. In this sense, Roitman^42^ described thermo-resistant species of *Crithidia*, and indeed, used this criterion to describe *Crithidia lucilae thermophila*.

The strain originally deposited as *Crithidia hutneri* was isolated and described by Isaac Roitman^42^ from the host *Cosmoclopius* [Supplementary data (Table III)] in Brazil and it was deposited as COLPROT018, for which ATCC has a duplicate ATCC 30818. Recently, we revised the taxonomy of several *Crithidia* isolates and proposed to join them to *C. thermophila*, which has the type strain the COLPROT054:^43^ *C. hutneri* (ATCC 30818, COLPROT018), *C. lucilae thermophila* (ATCC 30817, COLPROT054) and *Crithidia confusa* (ATCC PRA-346, COLPROT676).^43^ In addition, as part of the curation process, we decided to acquire the old preserved strains from ATCC to exclude sample mishandling, such as mixtures, mislabeling, etc. The “freshly” acquired strains from ATCC received new COLPROT numbers (*C. hutneri* - COLPROT688 and *C. lucilae thermophila* - COLPROT689). *gGAPDH* and *V7/V8* sequencing confirmed the previous finding, being possible to observe a monophyletic clade in Figure and by the high similarity in the BLASTn [Supplementary data (Table I)]. Furthermore, a recently isolated strain from COLPROT field expeditions (COLPROT703) was also assigned to this species, as well as an old preserved sample awaiting species designation (COLPROT056),^43^ which was isolated by Carvalho and Deane^19^ after the screening of 1000 *Zelus leucogrammus* for the presence of trypanosomatids. Several isolates were obtained and subsequently characterised. COLPROT056 is one of these isolates, which has been previously identified only up to the genus level.^44^

*Crithidia abscondita, Crithidia brachyflagelli, Crithidia brevicula, Crithidia insperata, Crithidia otongatchiensis* and *Crithidia permixta* - The deposits of the species *C. abscondita* (COLPROT677), *C. brachyflagelli* (COLPROT669), *C. brevicula* (COLPROT110 and COLPROT111), *C. insperata* (COLPROT670), *C. otongatchiensis* (COLPROT628 and COLPROT673) and *C. permixta* (COLPROT671) showed very low sequence divergence from the Genbank reference strains using the BLASTn tool and through the support of the topology observed by the phylogenetic analyses inferred by ML and BI. In this sense, these deposits had the taxonomic status confirmed [Figure and Supplementary data (Table I)].

***Herpetomonas* spp.**

The genus *Herpetomonas* harbors monoxenic trypanosomatids that present promastigote forms and another form, unique to the group, opisthomastigotes (rarely seen in insects and even in culture). However, promastigote forms can be observed in other genera, such as *Leptomonas* and *Phytomonas*. This generated ambiguities in the taxonomic classification of the genus *Herpetomonas*, mainly at the time when only classical taxonomy was performed.^45^ This prompted, Borghesan and collaborators to propose a complete revision of the genus *Herpetomonas*, since phylogenetic analyses revealed a polyphyletic group.^13^ ^,^ ^45^

*Herpetomonas costoris* and *Herpetomonas mirabilis* - The hapantotype of *H. costoris* is culture ATCC 30262, of which culture TCC 019E deposited at the TCC-USP,^13^ and COLPROT022 are duplicates. A similar situation of *H. mirabilis* occurred with the species *Leptomonas costoris*. This species was first described as belonging to the genus *Leptomonas* by Wallace,^46^ isolated from *Gerris comatus* (Hemiptera) in the United States. Borghesan^13^ reallocated the species in the genus *Herpetomonas* based on sequence analysis of *gGAPDH*, *SSU rRNA* and ITS. Our results for the *gGAPDH* gene corroborate this result and the COLPROT022 deposit was renamed as *H. costoris* [Figure and Supplementary data (Table I)].

The hapantotype for *H. mirabilis* is a culture deposited as ATCC 30263, of which TCC 301E deposited at the TCC-USP,^13^ and COLPROT024 are duplicates. This species was first described by Roubaud,^47^ isolated from a blowfly in Congo. In 1926, Wenyon reported trypanosomatids on blowflies from India, Italy and Sudan and described it as a new species of *Herpetomonas*. However, Wallace and Todd^48^ observed large flagellates in blowflies from Guatemala, naming them as *Leptomonas mirabilis*. The work of Borghesan^13^ has shown that this flagellate is a species of *Herpetomonas* and prioritise the description proposed by Wenyon,^49^ and the species was validated as *H. mirabilis*. Analysis of the *gGAPDH* and *SSU rRNA* genes corroborate the results obtained by Borghesan and the *L. mirabilis* deposit (COLPROT024) was also renamed to *H. mirabilis* [Figure and Supplementary data (Table I)].

*Herpetomonas elegans* and *Herpetomonas modestus* - The type material of *H. elegans* is culture TCC 1733 and of *H. modestus* is TCC 1444.^13^ *H*. *elegans* has three isolates obtained from *Ornidia obesa*, a syrphid. Here, we assign COLPROT211, a strain isolated from a Diptera and previously identified only at the family level, to this species [Figure and Supplementary data (Table I)]. Up to the moment, all isolates of this species are found in the family Syrphidae, which may indicate a host-specific interaction. Unequivocally, the sampling must increase to warrant further conclusions. On the other hand, COLPROT707 was identified as *H. modestus* [Figure and Supplementary data (Table I)] and it was isolated from a Muscidae, while the isolates previously described were all from the family Calliphoridae [Supplementary data **(Table III)**].

*Herpetomonas isaaci* - The type material of *H. isaaci* is culture TCC 266E and was described by Borghesan,^13^ a total of eighteen isolates were described, all of them from Diptera, mainly from the family Calliphoridae. During field expeditions done by COLPROT research team, we found two infected *Chrysomya megacephala* [Supplementary data (Table III)] with this tripanosomatid [Figure and Supplementary data (Table I)]. The activities were part of a teaching practical course performed in FIOCRUZ, Manguinhos campus (COLPROT710 and 711). In addition, we also isolated this species from feces of pigs in a peri-urban area of Rio de Janeiro (COLPROT682). The sampling, initially aimed at isolating *Trichomonas* and *Blastocystis*, revealed the presence of kinetoplastid flagellates, which may be a consequence of the high Diptera abundance. However, it is worth mentioning that kinetoplastids have been found consistently in feces from different animals, even in fresh released stools.^50^

*Herpetomonas muscarum* ^51^ - The isolates COLPROT013 and COLPROT229 refer to field isolation of trypanosomatids from tomatoes and their natural predators [Supplementary data (Table III)]. To our knowledge, this is the first time that *H. muscarum* ^51^ have been described in both the tomato fruit and its associated predator. The data reinforce the questioning of trypanosomatids’ host specificity.^52^ To sum up, COLPROT185 was isolated from a small rodent in Rio de Janeiro, Brazil. Although Dr RP Brazil and co-workers suspected from the beginning that the isolate was more related to *Herpetomonas* than to *Leishmania*, skepticism prevented proper taxa description^53^ (RP Brazil, personal communication). Finally, a previously unidentified strain (COLPROT081)^54^ isolated from a common weed in Brazil (previously assigned to *Phytomonas* sp.), COLPROT076 (previously described as *Phytomonas davidi*) and COLPROT033 (previously described as *Herpetomonas megaseliae*) presented very low sequence divergence to the type strain (COLPROT020) [Supplementary data (Table I)]. Thus, our results support the observations from Borghesan and colleagues^13^ for the synonymisation of *P. davidi* and *H. megaseliae* to *H. muscarum*. All the strains analysed presented very close sequences and are represented in the tree by COLPROT020, the type strain (Figure).

It is worth noting that the historical description of trypanosomatids, its preservation and taxonomic updates are full of controversies. Despite the classical and joyful disagreement among research groups, there is also a lack of trustable re-traceability of strains. In this sense, *Leptomonas pessoai* description is full of uncertainty, the deposit COLPROT276 had few records, but the work of Galvão^55^ that described *L. pessoai* was cited in the COLPROT records. By this information and by the DNA sequencing analysis, this available isolate, if actually related to the original description of Galvão, should be synonymised to *H. muscarum*, while the taxonomic validation of *L. pessoai* still waits confirmation.^11^

*Herpetomonas samueli* - The hapantotype culture is ATCC 30971 of which culture TCC 003E, deposited at the TCC-USP, and COLPROT074 are duplicates. According solely to *Leptomonas lactosovorans* Genbank sequences, this species should be considered a sister taxon of *H. samueli*.^13^ *L. lactosovorans* (COLPROT023) is a duplicate of ATCC 30970, the known culture of the isolate described by Manaia.^56^ DNA sequencing allowed the comparison between sequences in Genbank by using the BLASTn tool. This search returned that *L. lactosovorans* (COLPROT023) showed high identity with the *H. samueli* TCC 003E isolate for the *gGAPDH* target [Supplementary data (Table I)]. The phylogenetic analyses inferred by the ML and BI methods of this marker showed very robust topology values for this monophyletic clade that still includes the *Leptomonas wallacei* (COLPROT194) species (Figure).^57^ This last one, was isolated from *Oncopeltus fasciatus* raised in an insect laboratory colony [Supplementary data (Table III)].

*Herpetomonas samuelpessoai* ^*19*^ - *Herpetomonas anglusteri*, which is also available at ATCC (50304), was deposited at Coleção de Tripanossomatídeos (CT-IOC) in 1992 directly by Maurilio Soares (COLPROT059). It was isolated from a flesh fly of medical importance, either in forensic entomology context or as a myias is producing agent [Supplementary data (Table III)].^58^ According to the divergence profile acceptable for this species paratypes,^13^ we propose that this isolate COLPROT067 should also be listed as a paratype of the *H. samuelpessoai*,^19^ also available at TCC (005E) and ATCC (30252). Borghesan and colleagues^13^ suggested that *H. anglusteri*, deposited at TCC-USP, should be closely related to *C. fasciculata* (according to unpublished data from the research group).

***Kentomonas* sp.**

*Kentomonas sorsogonicus* - This new genus was described by Votýpka and collaborators,^59^ from a sample isolated from *Sarcophaga* sp. (Diptera), collected in the province of Sorsogon (Philippines). The flagellate was described as *Kentomonas* and only one species was identified: *K. sorsogonicus*. COLPROT received a deposit of this species (COLPROT696) by Dr Vyacheslav Yurchenko in 2015. This deposit presents a sequence identical to that deposited in GB (KM242072) by the analysis of the gene *gGAPDH* gene, which allowed the vouchering of the new species to the institutional collection [Figure and Supplementary data (Table I)].

***Lafontella* sp.**

*Lafontella mariadeanei* - This new erected genus was proposed to accommodate the old species *Herpetomonas mariadeanei* (TCC 004E)^5^ ^,^ ^60^. In the extensive revision of the *Herpetomonas* genus proposed by Borghesan,^13^ this species did not fit in the strongly supported clade composed of herpetomonads. In 2016, Yurchenko and colleagues proposed the new genus *Lafontella* to accommodate this species. Our data also support this reclassification and adds COLPROT062 as a type culture also available at COLPROT [Figure and Supplementary data (Table I)].

***Leptomonas* sp.**

The genus *Leptomonas* was designed based on morphological and host occurrence diagnosis, as all ancient described taxa in trypanosomatids. Except for a few examples, such as the phytomonads, which present a strong monophyletic clade. The analysis of single or multiple genes reveals a divergence that more likely reflects the limitations and resolution-power of the available methods for species cataloguing, than the actual phylogenetic divergence that can impact on evolutionary theories. The genus *Leptomonas* is clearly a phylogenetically artificial taxon with dubious types species, however, it is preferable to preserve this name rather the promoting a massive renaming of the numerous species placed in this genus over a long time.^61^ If one analyses strictly *gGAPDH* and *V7/V8* sequences, there are several species that overlap each other and some that better fit within the genus *Crithidia*, as well as the other way around.

*Leptomonas acus, Leptomonas spiculata* and *Leptomonas tarcoles* - COLPROT672, deposited by Dr VY Yurchenko as an isolate of *L. tarcoles*, shares identical *gGAPDH* and *V7/V8* sequences to Genbank deposits of *L. tarcoles* (EF546787 and EF546786, respectively), which is expected [Supplementary data (Table I)]. However, it is not possible to differentiate it from *L. acus* and *L. spiculata* solely based on *gGAPDH* and *V7/V8* (data not shown).^23^ ^,^ ^61^ Although ecological and biological data support differences between these isolates, there is strong phylogenetic evidence supporting a careful revision of the species currently allocated in this genus. Additional loci should be formally incorporated into the identification of these complex of cryptic species, since *gGAPDH* and *V7/V8* genes do not have resolution enough.

*Leptomonas jaderae* - COLPROT674, *L. jaderae* (34EC), was isolated from *Jadera obscura* (Rhopalidae) from Costa Rica [Supplementary data (Table III)], deposited by V Yurchenko at COLPROT in 2015 and was also deposited at the ATCC by the same research group. The sequencing of *gGAPDH* and V7/V8 confirmed the identity of the isolate deposited by V Yurchenko [Supplementary data (Table I)].^62^

*Leptomonas podlipaevi* - COLPROT344 was isolated from *Leptocoris triviattus* by Manaia in Campinas, São Paulo State, Brazil [Supplementary data (Table III)], and directly deposited in CT-IOC in 1999, as an isolate of *Leptomonas* sp.^63^ It showed low genetic divergence when compared with *L. podlipaevi* 59LI isolate for *gGAPDH* sequence (EU076604) and V7/V8 (EU079124) [Figure and Supplementary data (Table I)].^62^ Therefore, we assigned it as an isolate of *L. podlipaevi*.

*Leptomonas pyrrhocoris* - *L. pyrrhocoris* is a cosmopolitan species that mainly has the family Pyrrhocoridae as host^64^ [Supplementary data (Table III)]. COLPROT026 is a duplicate of ATCC 30974, which is the oldest isolate available in culture and represents the species designation. COLPROT026, COLPROT572 and ‘type sequences analyses’ from Genbank JN036651 (*gGAPDH*) and JN036653 (*V7/V8*) for *L. pyrrhocoris* ^64^ possess no nucleotide variation. COLPROT706, which has been recently isolated from *Dysdercus ruficollis* [Supplementary data (Table III)], shows little or no genetic divergence for the *gGAPDH* and *V7/V8* markers, respectively [Supplementary data (Table I)]. Collectively, this confirms the widespread distribution of this species around the world.^64^

*Leptomonas seymouri* - *L. seymouri*,^65^ the COLPROT277 deposit which is a duplicate of ATCC 30220, was isolated in 1959 from *Dysdercus suturellus* (Hemiptera: Pyrrhocoridae) in Florida, USA [Supplementary data (Table III)].^65^ The molecular identity of this deposit was confirmed when compared to the sequence of *gGAPDH* (KP717896) deposited in Genbank [Figure and Supplementary data (Table I)]. In addition, another strain isolated from *D. ruficollis*, collected in Rio Claro (Brazil) [Supplementary data (Table III)] and deposited by Dr Jan Votýpka of Charles University (Prague) (COLPROT705), was also attributed to this taxon [Figure and Supplementary data (Table I)].

***Phytomonas* spp.**

The type species of the genus *Phytomonas* is *Phytomonas françai*. This genus comprises plant trypanosomatids with promastigote forms. This first isolate received the name of *P. françai*, in honor of Carlos França.^66^ The name *Phytomonas* gained acceptance as the generic name for all trypanosomatids of plants, which led to the precipitated allocation of *Herpetomonas* and *Crithidia* species isolated from plants in the *Phytomonas* genus. Despite this, the genus is phylogenetically deﬁned as a monophyletic cluster according to *SSU rRNA* and *gGAPDH* genes, and the isolates that are divergent accommodate through molecular phylogeny in the genera *Herpetomonas*, *Crithidia* and *Leptomonas.* ^10^

The trypanosomatid *P. françai* was isolated in 1927 by Aragão from *Maniot palmata*. The currently available culture was isolated by Vainstein and Roitman^67^ in Brazil from *Maniot esculenta*, which was sent for analysis by Empresa Capixaba de Pesquisa Agropecuária (EMCAPA), of Espírito Santo State, Brazil, and is deposited as TCC064E. The original isolate described by Aragão^66^ is no longer available. COLPROT011 has the records to be a duplicate of TCC064E, however, its molecular characterisation did not validate it as such. COLPROT011 shared identical, *gGAPDH* and *V7/V8* sequences with COLPROT082, isolated from *Euphorbia pinea*, which is a duplicate of TCC306E and TCCEM1.Fr. These isolates are under *Phytomonas* phylogenetic lineage PhyD^10^ COLPROT082 was isolated from *E. pinea* and it was deposited as *Phytomonas* sp. Sequencing of *gGAPDH* and *V7/V8* reveals that it is an isolate of *Phytomonas* PhyD. The culture was donated by Marta Teixeira in 1994. In the original article that describes the strain isolation, Dollet and colleagues^68^ described the successful cloning of three samples: TCC 051E, TCC 053E and TCC EM1.Fr. COLPROT082 shares identical sequences of *gGAPDH* and *V7/V8* with these samples, which are all isolates from *E. pinea* from France. In addition, an isolate obtained from *Euphorbia hirta* from India also shares identical sequences. These isolates present ITS sequence divergence.^10^ COLPROT011 and COLPROT082 requires sequencing of additional loci in order to fully ascertain its position within *Phytomonas* PhyD group.

*Phytomonas serpens* - The first description of isolates from tomato fruits and a phytophagic hemiptera, *Nezara viridula*, was from Cape Peninsula, South Africa by Gibbs,^69^ who designated the isolate as *Leptomonas serpens*, which was not preserved. Therefore, considering the paradigm host/morphological stages, Podlipaev renamed it to *P. serpens*.^70^ Subsequently, all promastigotes isolated from tomatoes were described as *P. serpens*. Therefore, it would be expected as it occurred in several other genus or species group that a great heterogeneity should be encountered in the taxa. Interestingly, this is not the case and several isolates obtained by two distinct researchers in Brazil^71^ ^,^ ^72^ all fit within a closely related group. Indeed, all phytomonad formed a consistent phylogenetically related group, as previously described.^10^

COLPROT174, COLPROT186, COLPROT187, COLPROT188, COLPROT189 and COLPROT675 compose the “T series isolates from tomatoes”.^72^ The type isolate of this species has not been defined, not even in the recent molecular revision of the genus.^10^ To our records, the work of Jankevicius and colleagues^72^ is the oldest description of *P. serpens* isolation that still has the preserved samples in culture. The work describes a series of isolates, which were designated a number followed by the letter T. The most studied is 9T (COLPROT189, of which COLPROT174 is a duplicate). As such, we suggest that this isolate should constitute the type strain. In 2014, COLPROT received a strain, as part of a collaboration work with Dr Vyacheslav Yurchenko. This is also a duplicate from isolate 9T, and the molecular identification confirmed the identity [Supplementary data (Table I)]. The 9T was isolated from a tomato fruit in Rolandia, Paraná State, Brazil, the 10T isolate in Palmital, São Paulo State, Brazil, while 15T derives from an experimental infection of the insect. Although we can find citation to the 30T isolate in some old articles,^73^ we could not track its origin exactly.

Brazil and colleagues have been reporting the isolation of trypanosomatids from tomatoes and the feeding insect *Phthia picta* for many years, however without success in the isolation and in long-term culture of the isolates. After they succeeded in establishing stable cultures, the authors described the isolates as Trypanosomatidae sp. (COLPROT245 and COLPROT247), due to the lack of an available culture for comparison and to the uncertainties that morphological characterisation plus host origin information already posed.^71^ The present data clearly assigns COLPROT245 and COLPROT247 as strains of *P. serpens*, since virtually no sequence divergence was detected in both markers [Supplementary data (Table I)]. COLPROT080 was isolated from the fruit bergamot (*Citrus bergamia*) by Conchon and colleagues^74^ and deposited in 1992 in COLPROT by Marta Teixeira. This isolate had the ITS sequenced,^10^ which assigned it to the *P. serpens* PhyA group. However, the authors did not generate *V7/V8* or *gGAPDH* sequences. Here, we provided these sequences, which reinforce its classification within PhyA, and as such we propose it to be regarded as an isolate of *P. serpens* [Supplementary data (Table I)].^10^

COLPROT083 was deposited by Marta Teixeira in 1994. The phytomonad was isolated from the latex of *Jathropha macantha* and was classified within PhyA group, which contains the widely studied species *P. serpens*.^10^ However, the authors claim that the strain history is questionable; the strain is referenced to Burstein and Romero.^9^ ^,^ ^75^ Although Zanetti and colleagues^10^ decided not to make public a DNA sequence with a questionable history, here, we chose a different direction: make public all DNA sequences, and adding comments to the vouchered sequence. Then, other researchers can compare with their own isolates, or even the same vouchered species, which will greatly speed up taxonomic revisions.

The isolate COLPROT181 was deposited at COLPROT by Janckevicius in 1996. The isolate was described as *Herpetomonas macgheei*.^76^ It was named in honor of Robert Barclay McGhee, a major contributor to the knowledge on *Phytomonas*, and it represented the first report of a flagellate infection on gramineous crops, since it was isolated from *Leptoglos suszonatus*, which was feeding on Corn (*Zea mays*). Later, Borghesan^13^ indicated that this isolate should be reallocated to the *Phytomonas* genus, and recently, Zanetti and colleagues^10^ grouped it in the PhyA lineage, which is represented by *P. serpens*. According to our data, and the analysis of available sequences, we also strongly recommend it to be regarded as an isolate of *P. serpens*. COLPROT161 is a duplicate of the original isolate obtained from another researcher, A. Romeiro, which indicates that the available circulating cultures are indeed the same isolate. COLPROT161 and COLPROT181 share 100% identity with TCC 297E. Our suggestion to include all these isolates as strains of *P. serpens* comes from other strains grouping based in *gGAPDH* and *V7/V8*.^10^ ^,^ ^12^ ^,^ ^13^ ^,^ ^43^ However, it must be stressed that other markers, or even whole genome sequencing, can reveal interesting strain differences and intrinsic characteristics.

***Strigomonas* spp.**

The genus *Strigomonas*, as *Angomonas*, was validated by Teixeira and colleagues^12^ to allocate trypanosomatids harboring endosymbionts and presenting the choanomastigote form, but not related to the genus *Crithidia*. According to the authors, *Strigomonas* and *Angomonas* can be distinguished by kinetoplast morphology, kDNA molecular characteristics and phylogenetic analyses based on the SSU rRNA, ITS1 rDNA and gGAPDH genes.

*Strigomonas culicis* - The hapantotype is culture ATCC 30268, with duplicates at TCC 012E and COLPROT041. This species was originally described as *Trypanosoma* (*Herpetomonas*) *culicis* by Novy and colleagues,^77^ whom already questioned the uncertain boundaries of the morphology-based taxonomy: “it is in order to emphasise, as Leger has done, the fragile distinctions which exist between *Crithidia*, *Herpetomonas*, and *Trypanosoma*”.^77^ The cultures available nowadays refer to the isolate described by Wallace and Johnson,^78^ as a new genus, *Blastocrithidia*, isolated from *Aedes vexans*. It was recently reclassified as a member of the Strigomonadinae subfamily under the genus *Strigomonas.* ^12^ The latter was raised to accommodate trypanosomatids that contain an endosymbiotic bacterium, which is a trait of the group, as well as their monophyletic relatedness.^12^ An aposymbiotic strain was generated by prolonged antibiotic-treatment by Chang,^79^ and it is available at COLPROT034 and ATCC. Intriguingly, Teixeira and colleagues^12^ list ATCC 30257 as a duplicate of TCC012E, however, according to our records and ATCC catalogue, ATCC 30257 refers to the aposymbiotic strain [Supplementary data (Table I)].

*Strigomonas oncopelti* - The hapantotype culture is ATCC 12982, of which culture COLPROT055 is a duplicate, as well as TCC 043E. This specimen was originally described as *Herpetomonas oncopelti*.^36^ Later, the species was moved to the *Crithidia* genus,^11^ subsequently assigned to *Strigomonas*.^12^ Although the host has been described as uncertain in ATCC files, the original article unequivocally assigns the host as *Oncopeltus fasciatus*.^36^ The nowadays available cultures are originated from the ones kept by FG Wallace, which were subsequently donated to KP Chang, who performed experiments to eliminate, by antibiotic treatment, the endosymbiont^79^ (COLPROT019).

***Wallacemonas* sp.**

The genus described as *Wallaceina* was created to harbor a species (*Wallaceina inconstans*) that had choanomastigote and endomastigote forms and that was previously described as *Crithidia brevicula.* ^80^ In 2014, Yurchenko and colleagues redefined the *Wallaceina* genus based on the *18S*, *gGAPDH*, and SL sequences. These markers showed that the genus was polyphyletic:^81^ four species (*W. brevicula*, *W. inconstans*, *Wallaceina vicina* and *Wallaceina podlipaevi*) are indeed one single species that form a distinct clade, and were then moved back to the genus *Crithdia*, as *C*. *brevicula*, the original group name.^25^ In addition, the authors also proposed that the name *Wallaceina* should be abolished and the three species that remained in the genus be relocated to a new genus named *Wallacemonas*. This genus has as a type species *Wallacemonas colossoma*.^25^

*Wallacemonas collosoma* - COLPROT073 was originally described as *Leptomonas collosoma* by Wallace and colleagues,^26^ and later moved to the new erected genus *Wallacemonas*, which better suits the molecular phylogeny.^25^ COLPROT073 is a duplicate of ATCC 30261 and they represent the type species.

***Zelonia* sp.**

According to the need of taxonomic revision of the genus *Leptomonas*, recently, *Leptomonas costaricensis* ^82^ was reassigned to a new genus, *Zelonia*, based on the *SSU rRNA* and *gGAPDH* barcode sequences.

*Zelonia australiensis* and *Zelonia costaricensis* - Espinosa and colleagues analysed the genes *SSU rRNA* and *gGAPDH* and the results showed that *L. costaricensis* is very distant from the *L. pyrrhocoris* and *L. seymouri* species and therefore the new genus was created to accommodate this species: *Z. costaricensis*.^6^ The COLPROT has a deposit (COLPROT627), which was renamed to the new genus. Another species was described in this genus, *Z. australiensis*.^83^ This species has been described to accommodate trypanosomatids isolated from females of *Simulium* (Morops) *dycei*, (Diptera: Simuliidae) found in Australia, which form a sister taxon of the relocated species *Z*. *costaricensis*.^83^

*Zelonia dedonderi* (Dedet, Geoffroy and Benichou 1986) Boucinha and d’Avila-Levy n. comb - Molecular and morphological data indicate that *Herpetomonas dedonderi* ^84^ does not belong to the genus *Herpetomonas* and does not fit into the genus *Crithidia*, although they present the choanomastigote form in culture.^13^ ^,^ ^45^ The COLPROT has a deposit of *H. dedonderi* (COLPROT060) isolated from *Haemagogus janthinomys* (Diptera) in 1991 in French Guiana [Supplementary data (Table III)]. The analysis of the *gGAPDH* and SSUrRNA genes showed that the genetic divergence between *H. dedonderi* species deposited in COLPROT and the recently reclassified *Z. costaricencis* supports that *H. dedonderi* is more related to the genus *Zelonia*, suggesting its elevation for this genus and the reclassification as *Z. dedonderi*, n. sp [Figure and Supplementary data (Table I)].

*Zelonia* n. sp. - The COLPROT616 (CEU334) deposit was received in 2003 from Dr Philippe Grellier from the Nacional Museum of Natural History in Paris, France, a strain isolated from *Rasahus surinamensis* (Reduvidae), collected in Guyana [Supplementary data (Table III)]. Analysis of the genes *gGAPDH* and *V7/V8* showed a divergence which suggests that this isolate can represent a new species of the genus *Zelonia* in comparison with the old species of *L. costaricensis* [Figure and Supplementary data (Table I)].

New genera

Finally, there are three deposits, COLPROT100, COLPROT101 and COLPROT728, which do not fit in any previously described clade. COLPROT100 and COLPROT101 were isolated from *Psychodopygus ayrozai* (Diptera), by Toby Barret in Brazil in 1993 [Supplementary data (Table III)]. COLPROT728 was isolated from *Oncopeltus fasciatus* colony (Hemiptera) by Alexandre Romeiro in 1998. The colony was established by Dr N Ratcliffe, Swansea University-UK and further maintained by Dr Patricia Azambuja Penna, Instituto Oswaldo Cruz, Rio de Janeiro, Brazil. This insect colony is still maintained, which prompted us to screen *O. fasciatus* insects. As expected, the trypanosomatid infection was confirmed, and re-isolation of the trypanosomatid revealed that the molecular identity of this new isolate (COLPROT751 and COLPROT755) matched an old deposit done by Dr A Romeiro, which represented another isolate from the same host (COLPROT728). Both isolates present sequence divergence to all known trypanosomatids conceivable with the proposition of a new typing unit.

COLPROT100 and COLPROT101 showed closer identity with the species *Sergeia podlipaevi* ^85^ for the *SSU rRNA* gene. For the *gGAPDH* gene, the species that came closest was *Leptomonas tenua* ^86^ [Supplementary data (Table I)]. COLPROT728 showed similarity with the *Leptomonas jaculum* species^87^ for the *SSU rRNA* marker and similarity with the species *Kentomonas sorsogonicus* for the *gGAPDH* gene [Supplementary data (Table I)].

Although the species mentioned above have a closer genetic identity to the discussed deposits, the percentages of divergency do not support their synonymy. Figure shows the well supported topology by the concatenated analysis of both genes inferred by ML and BI and Supplementary data (Table I) the BLASTn values suggests that they belong to complete novel taxa that deserve full description in upcoming studies, which will include morphological characterisation, host interaction, behavior in distinct growth conditions and whole genome sequencing.

*In Conclusion* - Taxonomic changes and updates are followed by “non-taxonomists” researchers with skepticism. There are long delays in changes of species names, reassemble, joining or reclassification in publications. Here, we provided a list of validated species with traceable histories and associated DNA sequences (using BLASTn tool) together the ML and BI analyses. We thereby contribute to the overall taxonomy and biodiversity knowledge of trypanosomatids and provide support for non-taxonomist researchers to clearly depict the taxonomic and phylogenetic relatedness of each sample described. This allows to correlate new isolates to the previously described ones easily and quickly. In addition, we carefully update the taxonomic information in each Genbank submission, in accordance to what was described in the present report. It is well known by the scientific community that old or inaccurate taxonomic description of DNA sequences in Genbank reduce the pace of phylogenetic and taxonomic studies.

Taxonomic summary

Class KinetoplasteaHonigberg, 1963

Subclass Metakinetoplastina Vickerman, 2004

Order Trypanosomatida Kent, 1880

Family Trypanosomatidae Doflein, 1901

Subfamily Leishmaniinae Maslov et Lukeš in Jirků et al., 2012

Genus *Zelonia* Shaw, Camargo and Teixeira 2016

*Zelonia dedonderi* (Dedet, Geoffroy & Benichou 1986) Boucinha and d’Avila-Levy n.comb.

Synonyms: *Herpetomonas dedonderi* Dedet, Geoffroy & Benichou 1986

Type material: the culture IHAUGF/8 IICAYI13 deposited in the collection of the Department of Medical Protozoology of the London School of Hygiene and Tropical Medicine is the holotype, which COLPROT 060 is a duplicate.

Type host: *Haemagogus janthinomys* Dyar, 1921 (Diptera: Culicidae)

Type location: rain forest, area of FRG (52”35‘W/4”55“) near Montsinery, French Guiana

Diagnosis: DNA sequences from gGAPDH (DQ383650) and SSU rRNA (V7/V8) (DQ383648)

Remarks: This isolate was originally described as *Herpetomonas dedonderi* by Dedet, Geoffroy & Benichou 1986, and a duplicate of the type culture was deposited in COLPROT by Erney Camargo in 1991. Teixeira^45^ and Borghesan^13^ suggested that this species does not fit in the genus *Herpetomonas*. Phylogenetic analyses conducted in the present work indicated the closer relationship with the genus *Zelonia*.
